# Bio-diagnostic performances of microRNAs set related to DNA damage response pathway among hepatitis C virus-associated hepatocellular carcinoma patients

**DOI:** 10.1186/s43141-023-00537-2

**Published:** 2023-08-17

**Authors:** Sara M. Abdo, Wafaa Gh. Shousha, Amal Ahmed Mohamed, Mohamed Elshobaky, Mohamed Saleh, Mostafa Mohamed Abdelhamid Ali

**Affiliations:** 1 Biochemistry Division, Chemistry Department, Faculty of Science, Helwan University, Cairo, Egypt; 2 Biochemistry Department, National Hepatology and Tropical Medicine Research Institute, Cairo University, Cairo, Egypt; 3 Internal Medicine Department, Faculty of Medicine, Cairo University, Cairo, Egypt; 4 Internal Medicine department, National Hepatology and Tropical Medicine Research Institute, Cairo, Egypt

**Keywords:** HCC,HCV-related HCC, miRNA-23a, miRNA-203, miRNA-100-5p, miRNA-16, AFP, *In silico* HCC-microRNAs, Serum biomarkers

## Abstract

**Background:**

Up to date, a well-defined microRNAs (miRNAs) profile involved in hepatocellular carcinoma (HCC) pathogenesis remains indecisive. Thus, employing miRNAs for HCC diagnosis is demanded for early therapeutic interventions. We aimed to evaluate the usage of miRNAs set related to the SuperPath: miRNAs involved in DNA damage response pathway as effective biomarkers for HCV-related HCC diagnosis.

**Results:**

The study enrolled 97 patients with HCV-related HCC, 84 with hepatitis C virus (HCV), 97 with liver cirrhosis (LC), and 84 healthy individuals. Serum miRNA-23a, miRNA-203, miRNA-100-5p, and miRNA-16 were quantified using qRT-PCR experiments, AFP and routine LFTs were estimated *via* standard techniques. Pathway enrichment analysis along with the construction of miRNAs regulatory network were performed. With respect to healthy individuals, miRNA-203, miRNA-100-5p, and miRNA-16 were significantly downregulated in HCC, HCV, and LC groups, while miRNA-23a showed significant upregulation (*p* < 0.001). miRNAs exhibited significant correlations with AFP, ALT, AST, and albumin. Also, elevated levels of miRNA-23a were recognized in patients with multiple focal lesions and/or lesion size > 5 cm. Additionally, the diagnostic performance of miRNA-23a expression level at a selected cut-off value of 3.99 overtakes AFP, while expressions of miR-203, miRNA-100-5p, and miRNA-16 represent poor diagnostic outcomes.

**Conclusions:**

Keeping in mind the individual variability and high level of heterogeneity in HCC, our data revealed the diagnostic value of miRNA-23a expression in HCV-related HCC patients. Further extra *in silico* HCC-specific microRNAs sets are demanded in diagnosis.

## Background

Hepatocellular carcinoma (HCC) accounts for 90% of primary liver cancers [1] and it represents the third most common cause of death from cancer worldwide, with an increasing incidence expected in the next decades [2]. The major risk factors are chronic viral hepatitis B and C (HBV and HCV), alcohol abuse, primary biliary cirrhosis, xenobiotics, diabetes, non-alcoholic fatty liver disease, and genetic disorders such as hemochromatosis and α1-antitrypsin deficiency [3, 4], autoimmune hepatitis, PAPSS1 locus, hypothyroidism, glycogen storage disease (type Ιa and type Ιb) and acute intermittent [1]. Early diagnosis of HCC is crucial for improving the survival rate of patients. Early detection using serum alpha-fetoprotein (AFP) level is limited by its low sensitivity [5], so there is an urgent demand to develop new non-invasive biomarkers with high accuracy and feasibility.

Micro-ribonucleic acids (miRNAs) represent an abundant class of endogenous small RNA molecules of 20–25 nucleotides in length capable of regulating gene expression either by direct cleavage of targeted messenger RNAs (mRNAs) or by inhibiting translation through complementarity to targeted mRNAs at the 3\-untranslated regions (UTRs) [6]. Deregulated expression of miRNA has been linked to a variety of cancers, including HCC [7]. Differential expression of miRNAs between tumor and normal tissues as well as the stability of microRNAs in serum and plasma establish the role of miRNAs as non-invasive efficient biomarkers in cancer diagnostics [7].

A line of evidence supported that miRNAs are considered powerful biological target molecules for HCC diagnosis and/or therapy. Besides, these circulating miRNAs can modulate several downstream target genes and signaling pathways in HCC. However, little is known concerning the underlying mechanisms as well as the association of miRNAs profiles with the pathogenesis of HCC of various etiologies, in addition to the limited number of miRNA-based clinical trials [8].

The current study aimed to assess using of serum miRNA-23a, miRNA-203, miRNA-100-5p, and miRNA-16 as predictive biomarkers for early detection of HCV-induced HCC, in addition, to highlight the links of these miRs with other laboratory indices and tumor characteristics. These miRNAs were included as microRNAs related to the SuperPath “miRNAs involved in DNA damage response pathway” and were chosen according to the bioinformatics databases.

## Methods

The study included 362 subjects recruited from National Hepatology and Tropical Medicine Research Institute Cairo, Egypt, between July 2019 and December 2021. Participants were categorized into 84 healthy subjects, 97 patients with liver cirrhosis, 84 patients with HCV, and 97 patients with HCC. Informed consents were obtained from participants prior to their enrollment in the study and approved by the ethical committee for Human Subject Research at National Hepatology and Tropical Medicine Research Institute. Patients with viral infections other than HCV, alcohol abuse, primary biliary cirrhosis, xenobiotics, non-alcoholic fatty liver disease, genetic disorders, and other cancers or metastatic liver cancer were excluded. All patients were submitted to detailed history and clinical assessment. Liver cirrhosis was diagnosed based on history, clinical examination, laboratory findings, and abdominal ultrasonography. The severity of liver disease was assessed by the Child-Pugh score [9]. HCC was diagnosed by abdominal ultrasonography; abdominal triphasic computed tomography (CT), and serum AFP and confirmed to be HCC by histologic assessment of liver biopsy. Tumor characteristics were detected including tumor size, focal lesion number, site, and portal vein invasion. Tumor staging was performed according to the Okuda staging system [10]. Fasting blood samples were collected from all participants for analysis of serum alpha-fetoprotein, liver function tests, and prothrombin time (as International normalization ratio (INR)).

Total RNA was isolated according to the manufacturer’s protocols using RN easy mini kit (Qiagen, Valencia, CA, and the USA, Cat. 74134). For qRT-PCR experiments, quantification of Micro-RNAs was performed using TaqMan Gene Expression (Applied Biosystems, Waltham, Massachusetts, USA, Cat. # 4427975). RNAU6 was used as a housekeeping gene (endogenous control) in this study. The primers for miR-100-5p were F: 5’-GAACCCGTAGATCCGAACT-3’ and R: 5’-CAGTGCGTGTCGTGGAGT-3’, for miR-203 were F: 5' CACTCCAGCTGGGGTGAAATGTTTAGGACCA, R: 5' CTCAACTGGTGTCGTGGA, for miR-16 F: 5’TAGCAGCACGTAAATATTGGCG3’, for miRNA-23a, 5'-AUCACAUUGCCAGGGAUUUCC-3', for U6 were F: 5’-CTCGCTTCGGCAGCACA-3’ and R: 5’-AACGCTTCACGAATTTGCGT-3’ respectively. The amplification conditions were 95 °C for 10 min, followed by 40 amplification cycles of 95°C for 15 s and 60 °C for 60 s. The expression levels of miRNA-23a, miRNA-203, miRNA-100-5p, and miRNA-16 were normalized to the controls and the fold change of each miRNA was calculated using the 2^−ΔΔCt^ method.

Data were statistically analyzed using SPSS software (V23). One-way ANOVA was used for between-groups comparisons. Data are presented as mean ± standard deviation (SD). Correlations between the variables were analyzed using Pearson’s correlation coefficient. The diagnostic performances of studied miRs were evaluated via the Receiver operator characteristic (ROC) area under the curve (AUC) and the best cut-off values with their selected sensitivity, specificity, and positive and negative predictive values were calculated. *P* value < 0.05 was considered significant.

Bioinformatics analysis included (1) Enrichment and Gene ontology (GO): the biological processes and gene ontology of miRNAs were obtained according to David functional annotation and GeneAnalytics (https://geneanalytics.genecards.org/). Also, the gene set enrichment analysis in addition to the pathways involved from KEGG Reactome and WikiPathways were performed using the gProfiler online software tool (http://biit.cs.ut.ee/gprofiler/gost). The circulating miRNA-23a, miRNA-203, miRNA-100-5p, and miRNA-16 were involved in the DNA damage response pathway (Homo sapiens) [WP:WP1545]. More info can be available *via* the National Cancer Institute’s Clinical Proteomic Tumor Analysis Consortium (CPTAC) Assay Portal (https://assays.cancer.gov/available_assays?wp_id=WP1545). (2) MiRNAs regulatory network construction: the targets of miRNA-23a, miRNA-203, miRNA-100-5p, and miRNA-16 were predicted using miRTargetLink 2.0 databases (https://www.ccb.uni-saarland.de/mirtargetlink2).

## Results

Demographic and laboratory data are shown in (Table 1). The biochemical parameters aspartate aminotransferase (AST), alanine aminotransferase (ALT), albumin, bilirubin, and α-fetoprotein (AFP), were as expected, within the reference range for control subjects. Among the patient groups, ALT, AST, prothrombin concentration, and AFP were significantly elevated as compared to the healthy controls. Notably, there were no significant differences in AFP serum levels in HCV as compared to HCC and cirrhosis patients. miRNAs expression levels were dysregulated as serum miRNA-203, miRNA-100-5p, and miRNA-16 were significantly downregulated in HCC and HCV patients, while miRNA-23a showed significant up-regulation with respect to health subjects (*p* < 0.001).

**Table 1:** Demographic and laboratory data of all studied groups

Variables	Healthy controls (*n *= 84)	HCV (*n* = 84)	HCC (*n *= 97)	Liver cirrhosis (*n *= 97)
Age	48 ± 12	55 ± 8	59 ± 9	55 ± 8
Gender				
Male (%)	55 (65.48%)	68 (80.95%)	43 (44.32%)	42 (43.29%)
Female (%)	29 (34.52%)	16 (19.04%)	54 (55.67%)	55 (56.7%)
AST [U/l]	32.62 ± 6.38	36.56 ± 10.38	98.05 ± 74.6 *	69.35 ± 29.95 *
ALT [U/l]	30.92 ± 7.36	34.6 ± 8.81	58.55 ± 36.11 *	46.75 ± 24.4 *
Bilirubin [mg/dl]	0.77 ± 0.19	0.73 ± 0.21	8.63 ± 19.51 *	4.39 ± 3.98 *
Albumin [g/dl]	3.85 ± 0.21	3.73 ± 0.52	2.59 ± 0.55 *	2.34 ± 0.6 *
INR	0.98 ± 0.06	1.21 ± 0.31 *	1.47 ± 0.32 *	1.71 ± 1.01 *
α-Fetoprotein [ng/ml]	6.12 ± 0.21	9.99 ± 4.47	365.84 ± 66.79 *	157.58 ± 21.12 *
miRNA-23a	1.28 ± 0.95	3.653 ± 0.62*	8.189 ± 8.707*	2.144 ± 1.85*
miRNA-203	7.132 ± 8.986	5.09 ± 1.28*	3.34 ± 1.19*	4.81 ± 1.41*
miRNA-100-5P	3.52 ± 1.2	2.69 ± 0.51*	1.87 ± 4.33*	2.37 ± 1.0
miRNA-16	3.0 ± 0.79	2.56±0.62*	0.89 ± 0.49*	1.95 ± 0.63*

Data are expressed as mean ± standard deviations (SD), *HCC* Hepatocellular carcinoma, *HCV* Hepatitis C virus, *AST* Aspartate aminotransferase, *ALT* Alanine aminotransferase, *INR* International normalized ratio

^*^The mean difference is significant at *P* < 0.05

Micro-RNA-23a was directly correlated with serum AST, ALT, and AFP and inversely correlated with serum albumin. Contrariwise miRNA-203, miRNA-100-5p, and miRNA-16 represented vice versa correlations with the previous indices. Also, serum miRNA-203 and miRNA-16 were inversely correlated with miRNA-23a (Table 2).

**Table 2:** Correlations matrix among serum miRNAs and liver function indices

Variables		Albumin	ALT	AST	Bilirubin	AFP	miR-23a	miR-203	miR-16	miR-100-5p
miR-23a	*r*	− 0.17^**^	0.14^**^	0.17^**^	0.11	0.21^**^	1	− 0.14^**^	− 0.36^**^	− 0.01
	*p*	0.001	0.01	0.001	0.06	0.00		0.009	0.00	0.81
miR-203	*r*	0.16^**^	− 0.11^*^	− 0.12^*^	− 0.07	− 0.09	− 0.14^**^	1	0.15^**^	0.11^*^
	*p*	0.003	0.03	0.02	0.19	0.077	0.009		0.01	0.03
miR-16	*r*	0.48^**^	− 0.34^**^	− 0.44^**^	− 0.20^**^	− 0.31^**^	− 0.36^**^	0.15^**^	1	0.15^**^
	*p*	0.00	0.00	0.00	0.00	0.00	0.00	0.01		0.005
miR100-5p	*r*	0.17^**^	− 0.16^**^	− 0.21^**^	− 0.1	− 0.10^*^	− 0.01	0.11^*^	0.15^**^	1
	*p*	0.002	0.002	0.00	0.08	0.04	0.81	0.03	0.005	

*AST* Aspartate aminotransferase, *ALT* Alanine aminotransferase, *AFP* α-fetoprotein

Spearman’s correlation coefficient (*r*)

^**^Correlation is significant at *P* ≤ 0.01

^*^Correlation is significant at *P* ≤ 0.05

Regarding the Okuda staging system, 12.28% of HCC patients presented in stage I, 40.35% of HCC patients presented in stage II, and 47.37% of HCC patients presented in stage III. Imaging showed that all HCC occurred on top of cirrhosis, ascites were present in 82.46% of the HCC patients, and portal vein thrombosis was found in 17.54%. Focal lesions were single in 56.14% of cases, affected the right lobe in 56.14% of cases and their size ranged from 3.2 to 14 cm. When miRNAs were studied according to the focal lesion characteristics, it was found that miRNA-23a levels were elevated in patients with focal lesion size equal to or more than 5 cm, in patients with multiple focal lesions; and in Okuda stage III as shown in (Table 3).

**Table 3:** MicroRNAs levels in relation to tumor characteristics and Okuda staging

**Variable(s)**		*N*	miRNA-23a		miRNA-100-5p		miRNA-16		miRNA-203	
			Mean (range)	*P*value	Mean	*P* value	Mean	*P* value	Mean	*P* value
Size of focal	Less than 5 cm	11	2.11 (0.38–2.67)	*0.01*	1.55 (0.28–2.97)	*0.79*	0.92 (0.54–2.1)	*0.82*	3.52 (2.0–4.9)	*0.57*
lesions	Equal or more than	78	8.97 (2.85–55.57)		1.91 (0.06–43.0)		0.88 (0.12–3.65)		3.31 (0.09–5.4)	
	5 cm									
No. of focal	Single	54	3.66 (0.38–5.51)	*0.00*	1.22 (0.06–2.98)	*0.1*	0.96 (0.19–3.65)	*0.10*	3.42 (0.09–5.4)	*0.45*
lesions	multiple	43	13.87 (4.13–55.5)		2.68 (0.09–43.0)		0.79 (0.12–2.45)		3.24 (0.09–4.89)	
Portal vein	Present	17	5.7 (0.38–34.15)	*0.00*	1.35 (0.06–3.90)	*0.009*	0.88 (0.12–3.65)	*0.81*	3.36 (0.090–5.4)	*0.65*
thrombosis	Absent	80	19.9 (4.13–55.57)		4.34 (0.3–43.0)		0.91 (0.45–2.45)		3.22 (0.340–4.8)	
Okuda stage	Stage I	12	2.18 (0.38–2.85)	*0.00*	1.66 (0.28–2.97)	*0.53*	0.92 (0.54–2.1)	*0.77*	3.56 (2.0–5.3)	*0.32*
	Stage II	39	5.2 (2.92–12.85)		1.33 (0.060–3.9)		0.84 (0.190–1.98)		3.49 (0.09–5.4)	
	Stage III	46	12.29 (2.92–55.57)		2.38 (0.09–43.0)		0.92 (0.12–3.650)		3.15 (0.09–4.89)	

**Fig. 1:**
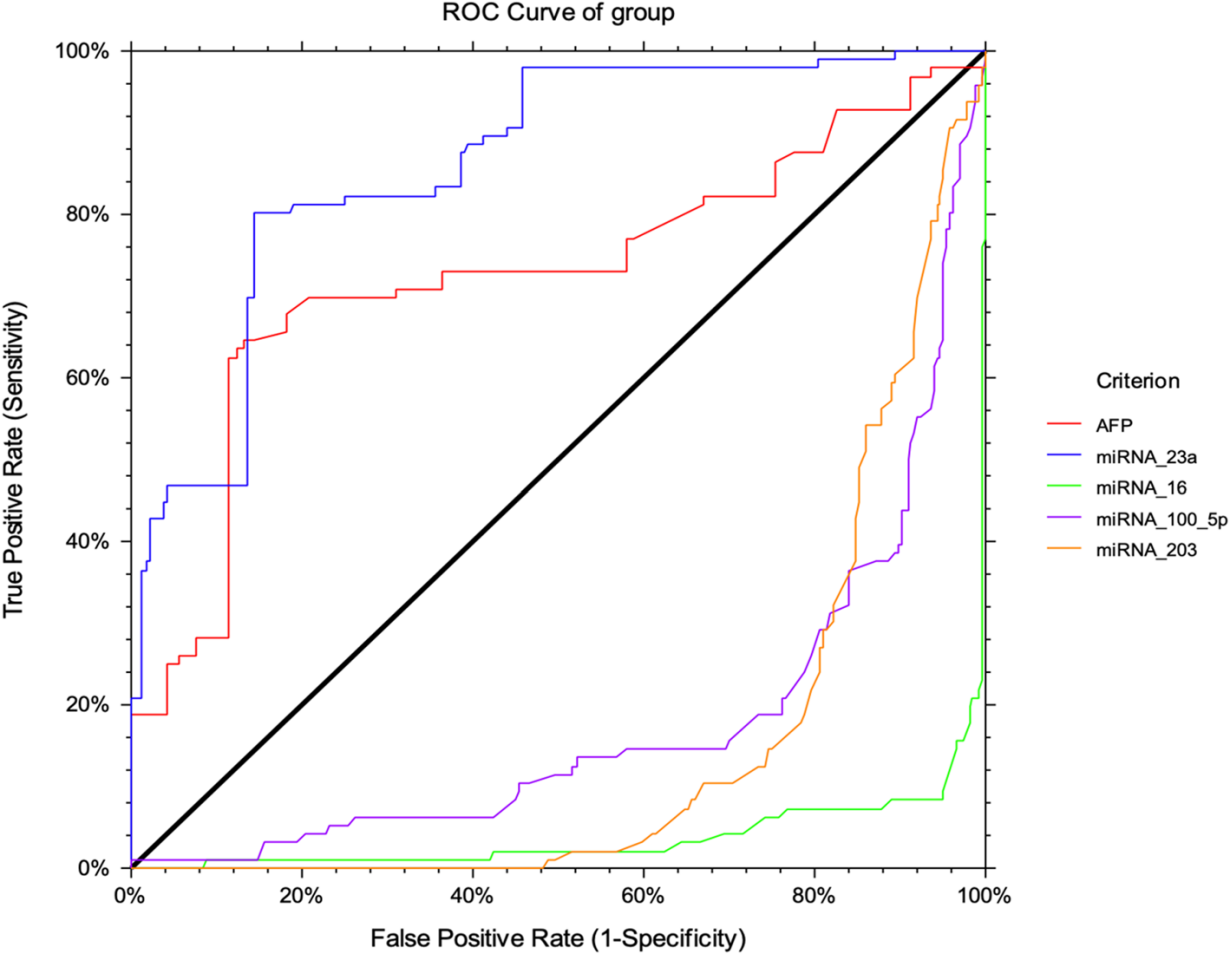
ROC curve analysis for miRNA-23a , miRNA-203, miRNA-100-5p and miRNA-16 versus α-fetoprotein

Receiving operating characteristic (ROC) analysis curves and the corresponding area under the curve were calculated for providing the diagnostic performances of microRNAs and AFP to discriminate HCV-induced HCC patients (Table 4). ROC curve analysis for microRNA-23a expression level represents a good diagnostic performance at a cut-off value of 3.99 (AUC = 0.86), which overtakes AFP, to differentiate healthy control and HCV patients from HCC patients (sensitivity about 80.21% and specificity 85.61%) (Fig. 1). On the other side, the expression levels of miRNA-203, miRNA-100-5p, and miRNA-16 represent poor diagnostic outcomes.

**Table 4:** Diagnostic performances of miRNAs and AFP among the studied groups

Variables	AUC	Cut-off	Sensitivity%	Specificity %	PPV	NPV	Accuracy
Healthy controls vs. hepatocellular carcinoma							
miRNA-23a	0.96 ^a^	2.31	97.94%	88.10%	90.48%	97.37%	93.37%
AFP	0.87 ^a^	12.00	73.20%	98.81%	98.61%	76.15%	85.08%
Healthy controls vs. HCV							
miRNA-23a	0.65 ^a^	2.81	96.43%	59.06%	41.75%	98.19%	67.78%
AFP	0.57 ^b^	3.20	96.43%	19.78%	26.64%	94.83%	37.57%
Hepatocellular carcinoma vs. non-malignant HCV							
miRNA-23a	0.86 ^a^	3.99	80.21%	85.61%	66.96%	92.24%	84.17%
AFP	0.72 ^a^	89.7	62.5%	88.64%	66.67%	86.67%	81.69%
Hepatocellular carcinoma vs. cirrhosis							
miRNA-23a	0.91 ^a^	2.31	97.94%	79.01%	71.43%	98.62%	85.61%
AFP	0.70 ^a^	89.70	61.86%	82.87%	65.93%	19.79%	75.54%

*AUC* area under the curve, *PPV* Positive predictive value, *NPV* Negative predictive value

^a^*P* < 0.001

^b^*P* < 0.01. The diagnostic performances of miRNA-203, miRNA-100-5p, and miRNA-16 were very poor and are not included in the table

**Fig. 2:**
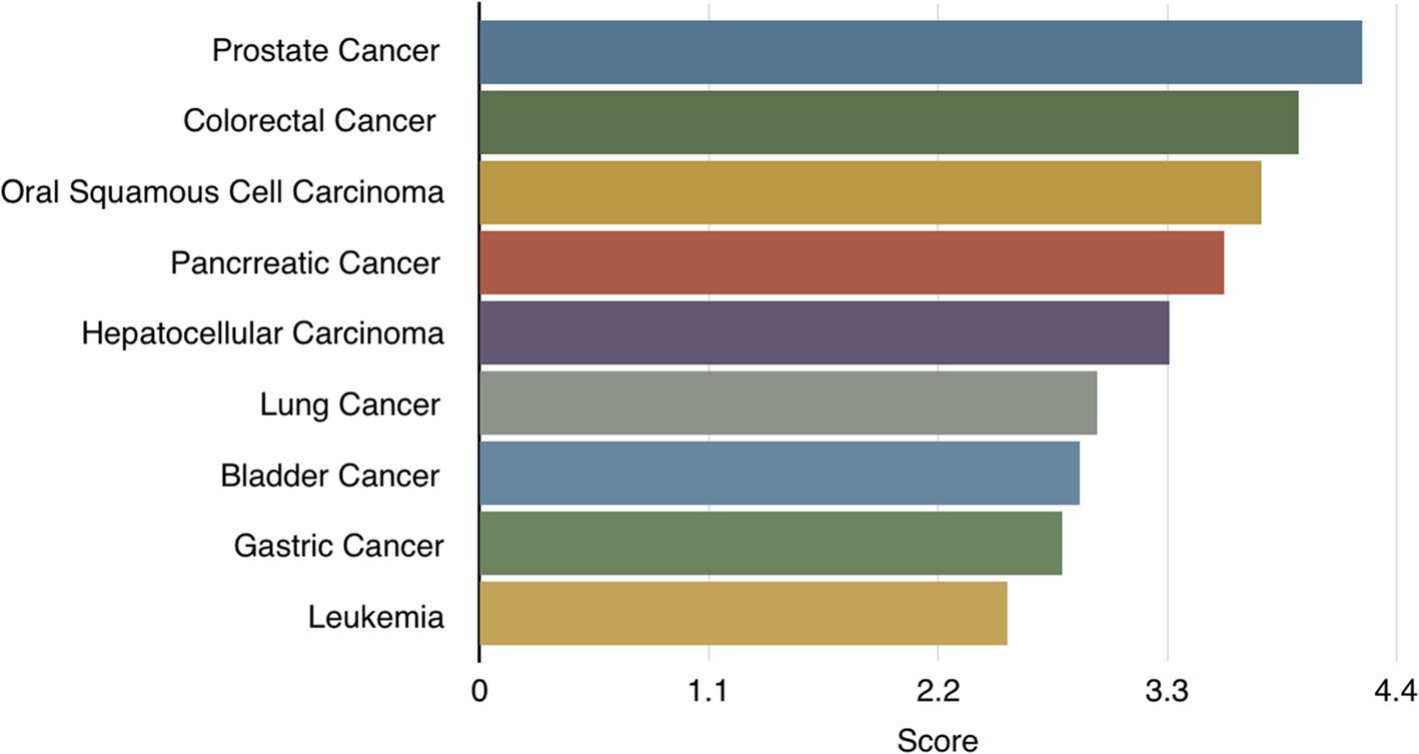
The Gene-disease relations of the studied miRNAs according to GeneAnalytics (https://geneanalytics.genecards.org)

**Fig. 3:**
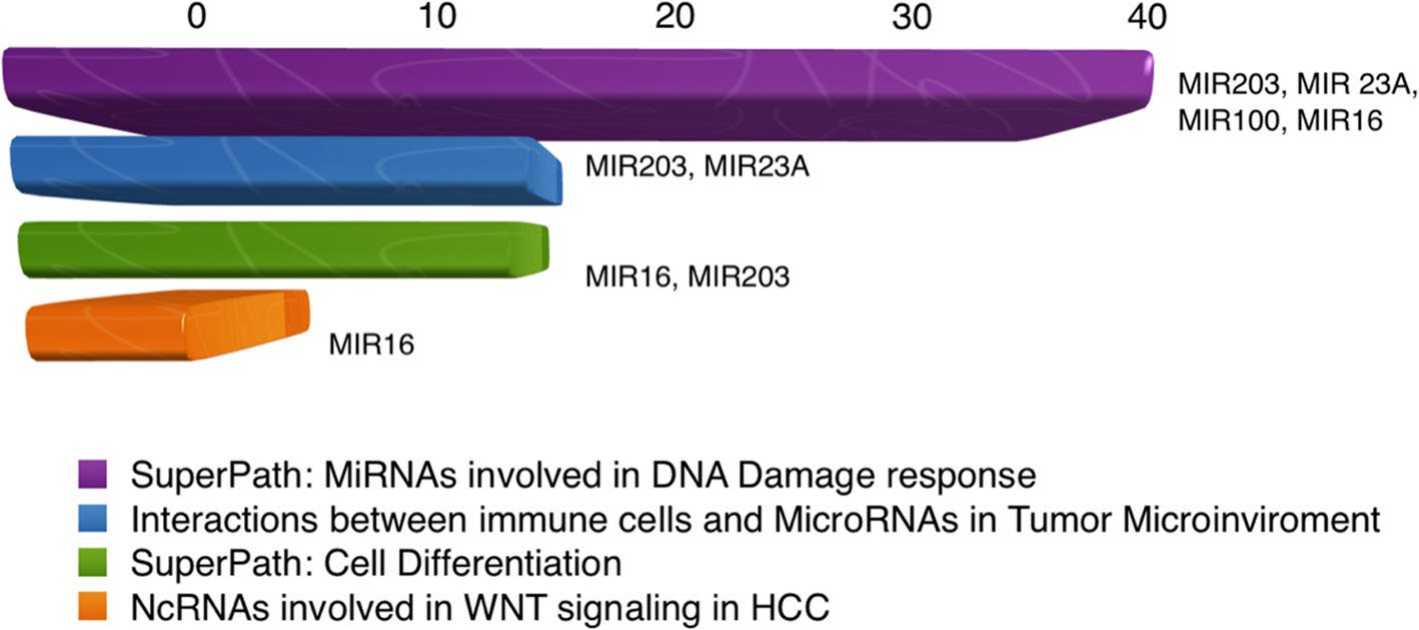
The main pathways related to the four studied miRNAs according to GeneAnalytics (https://geneanalytics.genecards.org)

**Fig. 4:**
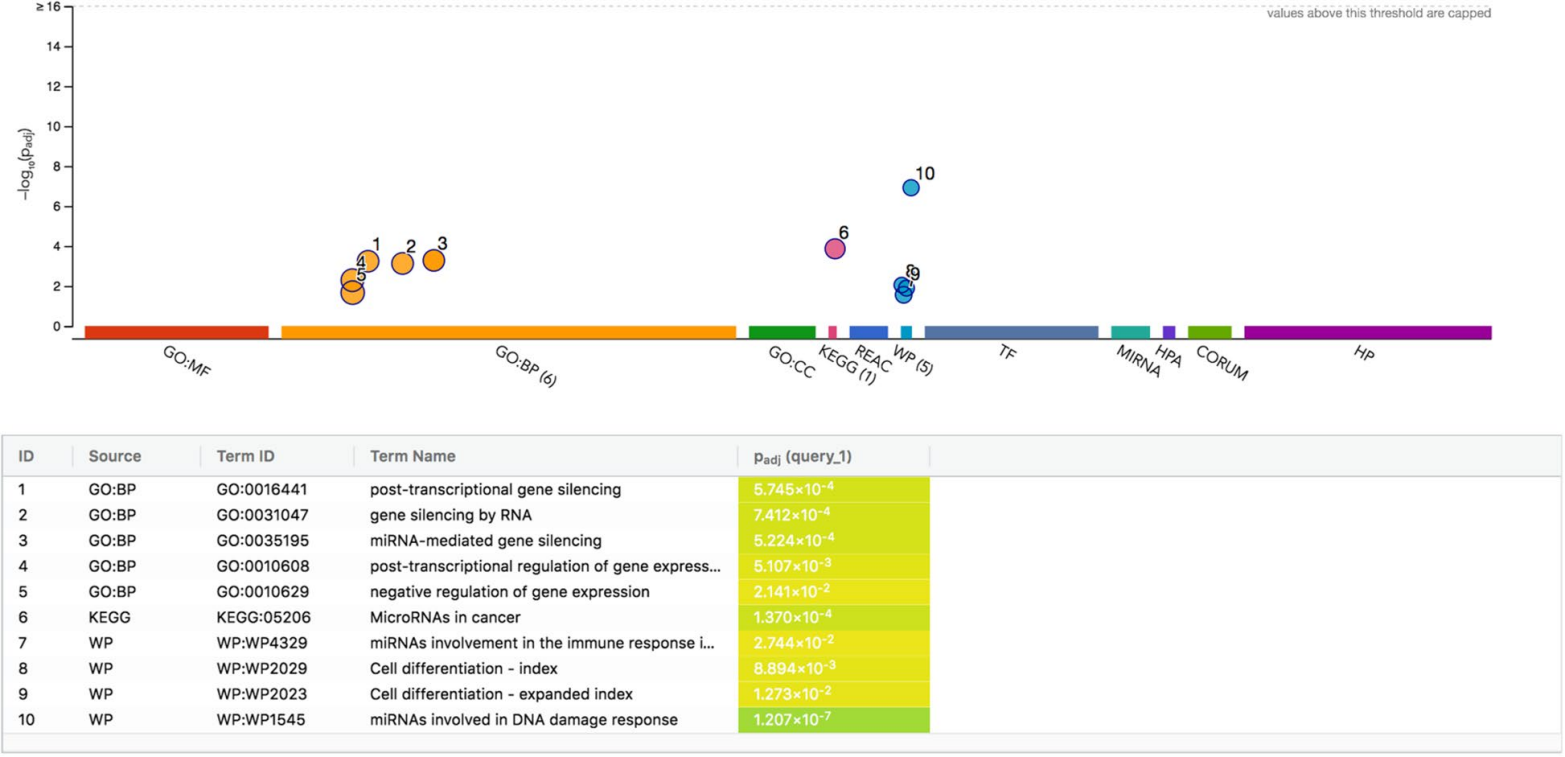
The gene ontology (GO) and pathways for the studied miRNAs. The biological processes and pathways from KEGG Reactome and WikiPathways according to g:Profiler (http://biit.cs.ut.ee/gprofiler/gost)

**Fig. 5:**
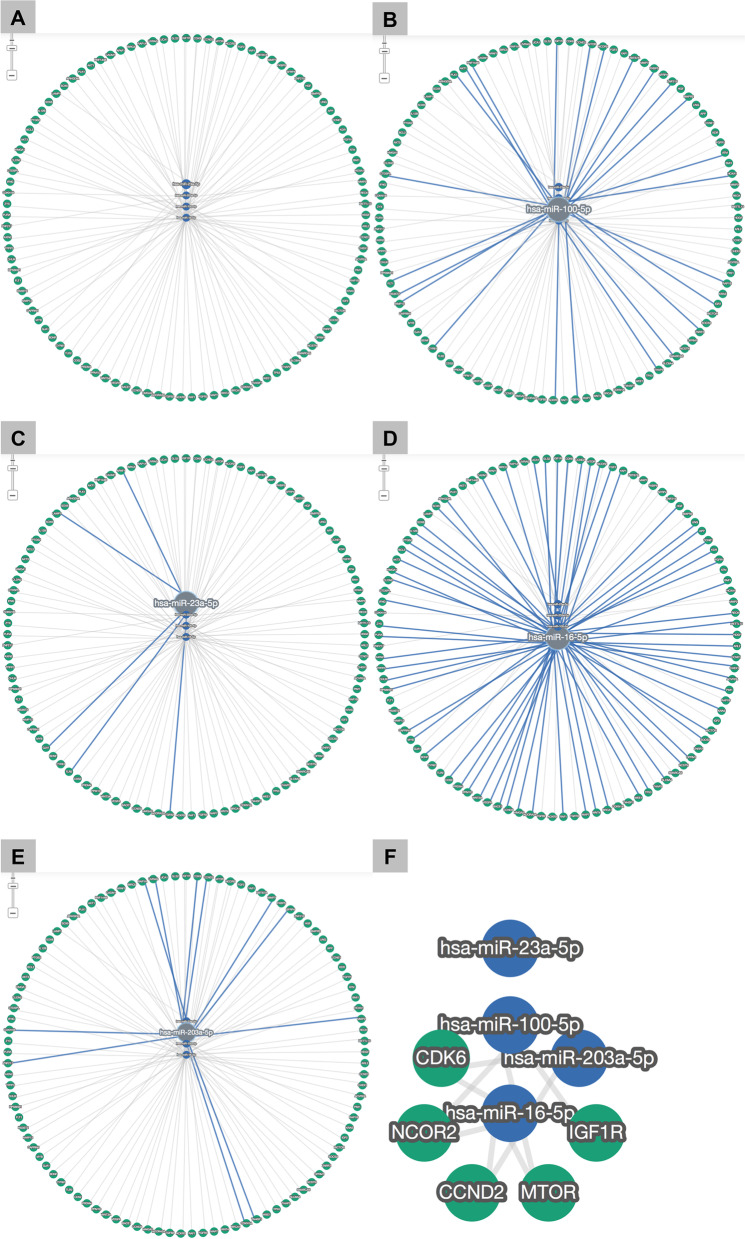
MiRNAs-target gene network analysis. **A** The studied miRNAs and their target genes network, only the strong validated miRNAs targets were used. **B**–**E** The target/target pathway of miR-100, miR-23a, miR-16, and miR-203 respectively. F: miRNAs and their strong validated target gene network with filtering option of minimum two shared targets, representing that target/target pathway of hsa-miR-100 were IGF1R, MTOR and NCOR2; for hsa-miR-16, CDK6 , CCND2, NCOR2, MTOR, and IGF1R; for hsa-miR-203, CCND2, and CDK6, while hsa-miR-23a did not share any common target genes with other miRs according to miRTargetLink 2.0 (https://ccbcompute.cs.uni-saarland.de/mirtargetlink2)

The GeneAnalytics disease-related outputs for the analyzed miRNAs set (hsa-miR-100, hsa-miR-16, hsa-miR-203, and hsa-miR-23a) indicated that they matched with ~ 88 diseases, mainly the prostate cancer and including their relations with HCC (Fig. 2). The main pathway related to all the studied MicroRNAs was the SuperPath: miRNAs involved in DNA damage response pathway, while miRNA-23a and miRNA-203 were involved also in interactions between immune cells and MicroRNAs in Tumor Microenvironment pathway (Figs. 3 and 4). The studied miRNAs set and their target genes network were represented in Fig.5.

## Discussion

Several body fluid miRNAs have been identified as cancer biomarkers that exhibit altered expression during tumor initiation and/or progression, aiding in disease diagnosis. However, Up to date, there is no standard reference miRNAs for HCC have been validated. We investigate the usage of a set of miRNAs, involved in the DNA damage response pathway, that could be used as effective biomarkers for HCC patients. Also, highlight reliable relations of these miRs with other laboratory markers and tumor characteristics. Along with highlighting more about these four miRs network.

Currently, numerous miRNAs profiling are employed to characterize cancer’s particular miRNA roles [11]. Dysregulation of miRNAs is considered one of the early events in tumorigenesis [1]. The pathogenesis of HCC comprises a multi-step and/ or stage process, aside from the influence of genetic and environmental factors. Equally, the circulating miRNA avenue has been explored in HCC pathogenesis, as several miRNAs represent their oncogenic and/or tumor suppressor behavior in cancer proliferation, in addition to their modulation of the apoptotic pathway [8, 12].

We have noticed an altered expression of serum miRNA-23a among 84 HCV and 97 HCC patients when compared to healthy individuals. In parallel, Li et al. [13] delineated the upregulation of miRNA-23a in HBV-positive HCC patients and suggested a role of miRNA-23a in the pathogenesis of HCC. In humans, the miR-23a gene is located at chromosome 19 and was transcribed as a part of the miR-23a–27a–24-2 cluster [14]. MicroRNA-23a has been involved in multiple roles including DNA repairing and cell proliferation, the initiation, progression, and metastasis, as well as the treatment response of human cancer [12]. Also, it was found that miRNA-23a down-regulates the expression of interferon regulatory factor-1 in HCC cells [15].

Regarding the role of miRNA-23a in hepatocarcinogenesis, the activation of IL6/Stat3 signaling in the liver mediated the up-regulation of miRNA-23a expression. Then, miRNA-23a directly targets Glucose-6-phosphatase (G6PC) and peroxisome proliferator-activated receptor-gamma coactivator 1-alpha (PGC-1α), to decrease the protein expression and promote carcinogenesis in the liver [16]. Notably, the reduction in G6PC is likely to result in concomitant accumulation of G6P. Alternatively, the tumors could metabolize excess G6P using the hexose monophosphate (HMP) shunt pathway and produce ribose-5-phosphate which is then used in nucleotide synthesis. This enhanced production of the key building block of nucleic acids is probably an important means of meeting the basic requirement for rapid cell division and growth of tumors. Moreover, the increased utilization of glycolytic and HMP shunt pathways in HCC, depending on the extent of accumulation of G6P due to block in gluconeogenesis, may contribute to the survival of the tumor cells under hypoxic conditions [17].

In the current study serum miRNA-203 was down-regulated in HCC, HCV, and liver cirrhosis patients with respect to normal individuals. Uniformly, it was reported that HCC patients with lower levels of miRNA-203 had increased cancer recurrence and shorter survival. This supports the notion that low expression of microRNA-203 is a significant predictor of poor prognosis in HCC patients who underwent LT [18]. Similar results indicated the downregulation of microRNA-203 in HCC patients in comparison to healthy control [19]. As a tumor suppressor miRNA, miRNA203 has been reported to target signal transducer and activator of transcription -1(STAT1) [20]. Additionally, miRNA-203 has been implicated in cell-cycle regulation and induction of apoptosis. The alteration of microRNA-203 expression affects the expression of survivin, where lower expression of microRNA-203 causes a corresponding increase in the expression of survivin and may promote HCC proliferation. The reason for the low expression of microRNA-203 in HCC tissue [21].

He et al. [22] have reported that miR-100-5p was down-regulated in HCC based on a total of 1,258 samples of clinical parameters compared with normal miR-100-5p expression. Likewise, our data of reduced expression of serum miRNA-1005p in HCV, HCC, and liver cirrhosis patients versus healthy individuals. miR-100 downregulation is correlated with progressive pathological features and poor prognosis in patients with HCC [23]. On the contrary, other studies found that circulating microRNA miR-100 was up to 2-fold up-regulated in primary liver nodules. This may indicate that miR-100 exerts an oncogenic function by the subsequent down-regulation of one or more tumor suppressor genes [24]. Serum miR-100-5p is aberrantly expressed and functions in many human cancers by regulating multiple cell processes, such as cell cycle, proliferation, differentiation, migration, invasion, and apoptosis by targeting Polo-like kinase 1 (PLK1), which plays an important role in the initiation, maintenance, and completion of mitosis [25–27].

Previous studies have indicated decreased tissue expression levels of microRNA-16 in HCC patients [28, 29]. An initial report describing the potential role of microRNA-16 in chronic lymphoma leukemia11 was followed by studies showing that it is frequently absent or down-regulated in chronic lymphoma leukemia and solid tumors. These data led to the hypothesis that microRNA-16 functions as a tumor suppressor. Proteomic and transcription analyses have revealed several microRNA-16 target genes, including B cell lymphoma 2 (BCL2), myeloid cell leukemia-1 (MCL1), Cyclin D1(CCND), and Wnt Family Member 3A (WNT3A) which are involved in cell growth, the cell cycle, oncogenesis, tumor suppression, and anti-apoptosis. The miR-16 plays an essential role in apoptosis by targeting BCL2 and the caspase signaling pathway [30].

A lot of miRNAs are strongly related to the modulation of HCV infection and replication. The dysregulation of multiple miRNAs due to HCV infection is accomplished through different pathways, including the cell cycle, lipid metabolism, and immune response. Target genes, like PPARG and fibronectin 1 (FN1), stearoyl-CoA desaturase (SCD), and CREB1, were found to be differentially expressed [8]. Many miRNAs patterns shifted to the normal values in the case of viral infection treatment, and miRNA expression in HCV patients treated with sovaldi showed improvement and reached nearly normal values [31]. In accordance with World Health Organization, the elimination of HCV infection is of international interest goal by 2030 [32]. We have noted an accepted performance of microRNA-23a to differentiate HCV patients from healthy controls.

With unsatisfactory results, the routinely used diagnostic tools for HCC detection are serum alpha-fetoprotein and liver ultrasonography (US) [33]. Alpha-fetoprotein can be elevated in other diseases and can be used to follow patients treated for HCV [34]. However, in HCC, the range of specificity and sensitivity of AFP are 76–94% and 39–65%, respectively, depending on the cut-off value [1]. Comparably, we reported a good diagnostic performance for microRNA-23a that surpasses AFP in HCV-related hepatocellular carcinoma patients. As well in this study miR-23a levels were significantly elevated in patients with focal lesions of 5 cm or more in size, patients with multiple focal lesions, and Okuda stage III versus patients with less advanced HCC disease. However, according to The Cancer Genome Atlas (TCGA) database, the higher expression level of miR-23a in HCC predicted a poorer overall survival [16, 35].

MicroRNAs could target various mRNAs *via* direct binding into their 3'-untranslated region, causing suppression of the gene expression. However, in particular cancers, the cell fate decided by the miRs expression could be in the opposite direction as the expressions of some target proteins were defective [16]. Further, microRNAs may possess the ability to target oncogenes and/or tumor suppressor genes that contribute to its dual activity role as a tumor suppressor and oncomirs through miRNA-long non-coding RNA-transcription factors and protein-protein interaction (lncRNA-TFs-PPI) Crosstalk. In this study, according to the genes/microRNAs network, it was recognized that IGF1R, MTOR, and NCOR2 were the common shared targets for miR-100 and miR-16; for miR-16 and miR-203 the common shared targets were CCND2 and CDK6; no common shared target for miR-100 and miR-203; while miR-23a didn't share any common target genes with other miRs. These testified target genes participated in various biological signaling pathways including cellular proliferation, differentiation, metastasis, invasion, and angiogenesis together with apoptosis.

## Conclusions

Nowadays, assorted miRNAs have been addressed the modulation of biological processes in HCC development. The usage of HCC-specific miRNA profiles that are involved in the initiation along with the progression of HCC, could provide effective biomarkers and/or improved therapeutic targets. Herein our results denoted that (1) serum miRNA-23a, miRNA-203, miRNA-100-5p, and miRNA-16 showed altered expression among cirrhotic, HCV, and HCC patients versus the healthy individuals. (2) serum miRNA-203 and miRNA-16 were inversely correlated with miRNA-23a and all miRs exhibited significant correlations with other indices such as AFP, ALT, AST, and albumin. (3) Among the studied microRNAs related to the DNA damage response pathway, miRNA-23a could serve as a screening biomarker with a suitable diagnostic behavior, which overtakes AFP, for early detection of HCC patients (who are previously infected with HCV). Finally, future studies should predict new *in silico* targets for microRNAs plus consider more HCC-specific microRNAs sets to be validated in diagnosis.

## Data Availability

Not applicable.

## Abbreviations

MiRNAs: Micro-ribonucleic acids
HCC: Hepatocellular carcinoma
HCV: Hepatitis C virus
LC: Liver cirrhosis
AFP: Alpha-fetoprotein
CT: Computed tomography
INR: International normalization ratio
SD: Standard deviation
ROC: Receiving operating characteristic
GO: Gene ontology
AST: Aspartate aminotransferase
ALT: Alanine aminotransferase
G6PC: Glucose-6-phosphatase
PGC-1α: Peroxisome proliferator-activated receptor-gamma coactivator 1-alpha
HMP: Hexose monophosphate
